# Supplementary figures and images for: Multiplex PCR Approach for Rapid African Swine Fever Virus Genotyping
Source: Viruses. 2024 Sep 13;16(9):1460. doi: 10.3390/v16091460 (PMC11437429; doi:10.3390/v16091460)

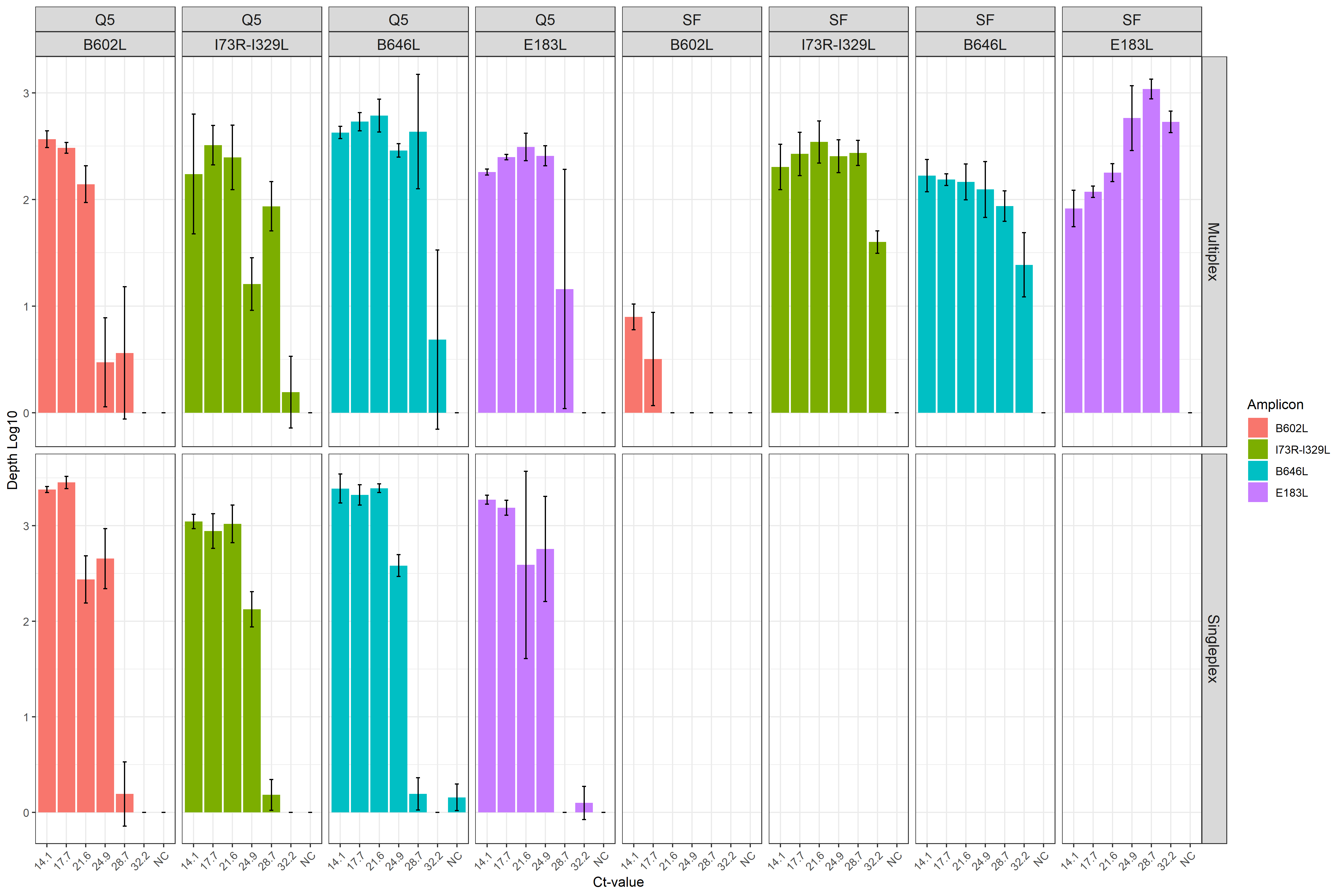

Supplement: Supplementary file 1 [file viruses-16-01460-s001.zip › viruses-3034302-supplementary.tiff]
